# The fellowship of regulatory and tissue-resident memory cells

**DOI:** 10.1038/s41385-021-00456-w

**Published:** 2021-10-04

**Authors:** Leandro Barros, Cristina Ferreira, Marc Veldhoen

**Affiliations:** Instituto de Medicina Molecular João Lobo Antunes, Faculdade de Medicina da Universidade de Lisboa, Av. Professor Egas Moniz, Lisbon, 1649-028 Portugal

## Abstract

T cells located in non-lymphoid tissues have come to prominence in recent years. CD8^+^ tissue-resident memory (Trm) cells are important for tissue immune surveillance, provide an important line of defence against invading pathogens and show promise in cancer therapies. These cells differ in phenotype from other memory populations, are adapted to the tissue they home to where they found their cognate antigen and have different metabolic requirements for survival and activation. CD4^+^ Foxp3^+^ regulatory T (Treg) cells also consist of specialised populations, found in non-lymphoid tissues, with distinct transcriptional programmes. These cells have equally adapted to function in the tissue they made their home. Both Trm and Treg cells have functions beyond immune defence, involving tissue homeostasis, repair and turnover. They are part of a multicellular communication network. Intriguingly, occupying the same niche, Treg cells are important in the establishment of Trm cells, which may have implications to harness the immune surveillance and tissue homeostasis properties of Trm cells for future therapies.

## Introduction

Lymphocyte populations have been studied during their development in primary immune organs, during their seeding, maintenance, circulation and after encounter of cognate antigen in secondary lymphoid organs. Naïve T cells circulate in large numbers, and harbour many antigen-specificities. The memory compartment, consisting of antigen-experienced cells, is composed of antigen-specific cells that contain many more clones with the same specificity. Immunological memory is one of the hallmarks of the adaptive immune response. However, the generation of memory T cells, particularly in which stages of the immune response it takes place and its molecular mechanisms, remains incompletely understood. Although there is increasing evidence that this heterogeneity exists in CD4 T cells, the majority of studies have focussed on CD8 T cells and we will largely limit ourselves to these.

Antigen-experienced cells may populate peripheral tissues, are poised for activation and effector function upon stimulation and show distinct metabolic states^1–3^. Functional differences between naïve and memory T cells account for swift and better immune protection. In part, this is explained by increased antigen-specific T cells in the memory compartment. Functional differences, such as the more rapid response, make additional important contributions to the level of immune protection^4^. The response will depend on the site of infection and the nature of the pathogen. The memory response is tailored to provide optimal protection against the re-invading pathogen thereby limiting the risk for host pathology.

Tissue immune responses have largely been studied in context of an ongoing inflammation. However, it has become clear that lymphocytes are a critical part of tissues during health. The discovery of innate lymphoid cells (ILC) gave impulse to the study of lymphoid cells seeded in tissues where they form an integral part of the fabric^5^. Amongst tissue-integrated immune cells are a large population of T cells. Those expressing the γδ T cell receptor (TCR) have long been known to have a distinct tissue distribution^6^. Especially at barrier sites, the top layers of the skin and the small intestine, these compartments are occupied by γδ T cells. This same niche is increasingly filled during life by CD8-expressing tissue-resident memory T (Trm) cells derived from antigen-activated circulating T cells^6,7^. Memory T cells are not a homogeneous population, with subsets specialisation for optimal immune surveillance and protection. Trm cells are an important component of the heterologous immunological memory pool, phenotypically and transcriptionally different from circulating memory T cells. The importance of Trm cells is derived from their unique location in the tissues, potent effector functions and bystander activity.

Inflammation is a risky state for an organism, away from the optimal status quo. Hence, inflammation is licenced by a tightly controlled sequence of events with many checks. After initial innate sensing of pathogens, it involves CD4-expressing helper T cells that boost and orchestrate the appropriate type of immune response and manage its level of activity. A critical component are CD4^+^ Foxp3-expressing regulatory T (Treg) cells. In addition, Treg cells are a heterogeneous population and home to a variety of different non-lymphoid tissues^8^. Initially considered “suppressor” T cells, reducing the activity of other T cells, they are now recognised to control the response of many cell types, including the generation of Trm cells^9^. This review will discuss the generation of tissue-resident memory cells in light of the role Treg cells may play in the formation of these cells.

### CD8 memory T cells

Effector memory T (Tem) cells share many characteristics with recently activated effector T cells and dynamically patrol the host, via circulation and the entering of many tissues, for remnants of the previously encountered pathogen or for signs of reinfection. CD62L and chemokine C Receptor (CCR)7 play important roles in controlling T cell movement towards secondary lymphoid organs (SLO). To facilitate free circulation in tissues, Tem cells do not express these surface factors^10^. Central memory T (Tcm) cells predominantly home in SLO, and express high levels of CCR7 and CD62L. Although these subsets were identified based on expression of surface molecules and location, they differ in functional capacities, regulated by transcription factors enabling the expression of receptors and signalling molecules. Mouse studies have shown that Tem cells are enriched outside the SLO, with Tcm cells dominating the SLO where they persist and can be reactivated^4^.

Due to their circulating behaviour, Tem cells were thought to be the main memory T cell subset to survey the peripheral tissues. However, a more recently defined memory subset are Trm cells. As their name implies, their defining feature is their commitment to remain in their tissue of residence, where they encountered the pathogen, and not circulate through the blood stream or SLOs. Trm cells do not express CCR7 or CD62L, but most express CD69. CD69 is an early activation marker for lymphocytes in SLO, it antagonises the egress receptor S1P receptor 1 (S1PR1), thereby preventing cells from leaving tissues, receiving optimal stimuli prior to downregulating CD69 and their release into the circulation. High expression of CD69 on Trm cells is constitutive and maintains their tissue residency^11,12^, but can vary per organ^13^. In addition, the integrins CD103 and CD49a can mark Trm cells, although Trm cells without CD103 expression have been detected in tissues^14,15^, while CD49a may reflect Trm cell activation status, provide motility and survival signals^16,17^. The integrin αE (CD103), which pairs with β7, is normally present on Trm cells residing at epithelial barriers, such as the skin and the intestine. It has an adhesive function through its binding to the epithelial cell marker E-cadherin, promoting retention of T cells in the tissues and facilitating their surveillance function^17^. It also potentiates T cell killing by directing cytolytic granule polarisation and exocytosis. In addition, most Trm cells express CXCR3 and have intermediate or low expression of CX3CR1^15,18,19^.

A distinct transcriptional programme drives surface markers indicative of T cell location and function. Trm cells, opposed to other memory CD8 T cell subsets, express low levels of Tbox protein expressed in T cells (Tbet) and Krüppel-like Factor 2 (KLF2), while Eomesodermin (Eomes) is absent^11,20^. Instead, Trm cells express high levels of arylhydrocarbon receptor (AhR) in gut and skin, Hobit and Blimp-1^7,21^. Hobit and Blimp-1 are two transcription factors playing a role in the establishment of tissue residency. Using murine models deficient for either or both showed these factors to cooperate to repress several genes involved in tissue egress from tissues like the skin, the small intestine and the liver^21^. This is not, however, a universal mechanism as only Blimp-1 is essential for the generation of Trm cells in the mouse lung^22^, and neither is differentially expressed by human lung Trm cells^23^. On the other hand, Runx3 has been associated with T cell residency programmes in both mice and humans^23,24^.

The important aspect of Trm cells in host protection is their location, especially in peripheral tissues such as the skin, intestine and lungs, but also the liver, which are directly and indirectly exposed to invading pathogens. Upon activation, Trm cells secrete cytokines and chemokines, promoting the recruitment of immune cells^25–27^. Furthermore, due to cytotoxic properties, they can directly kill infected cells, and thereby contribute to controlling pathogen-load^16,28^. Collectively, the activities of Trm cells provide protection against different types of invading pathogens, such as viruses^18,26,29^ and intracellular parasites^30,31^ in different tissues. These results make the generation of Trm cells at pathogen entry sites an increasingly interesting strategy for vaccine development.

### Treg subsets

**Fig. 1:**
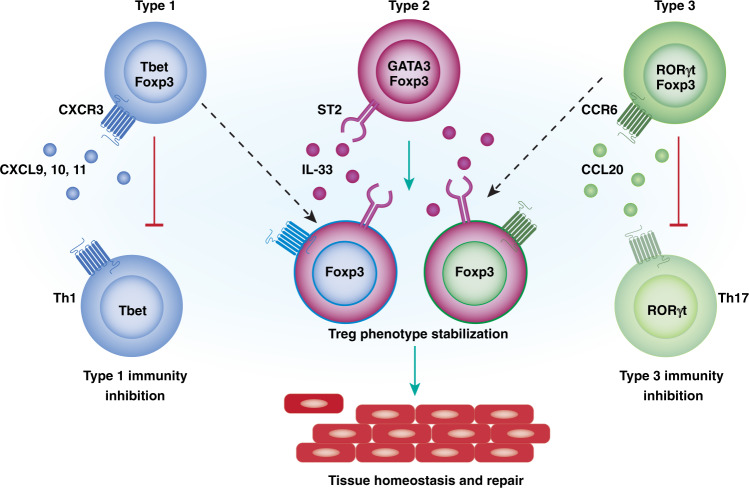
Overview of three main recognised Treg subsets and their potential role in immunity. Three main types of Treg cells are recognised, type 1 displaying features in common with Th1, such as the transcription factor Tbet and the chemokine receptor CXCR3. Type 3 Treg cells share a transcriptional programme with Th17 cells, such as expression of RORγt and CCR6. Each Treg subset can modulate respective immune responses (red lines). Type 2 Treg cells are characterised by expression of GATA3 and ST2 amongst others. There is limited data if this subset is involved in modulating type 2 immunity. ST2 may have a role in stabilising effector Treg cells and in tissue repair and homeostasis. In addition, type 1 and 3 Treg cells may express ST2 and may potentially show a convergence with type 2 Treg cells (dashed lines) and provide a similar role in tissue repair (green arrows).

Treg cells, characterised by the expression of the transcription factor forkhead protein 3 (Foxp3), are a subset of CD4 T cells involved in controlling immune response, thereby reducing pathology^32^. Treg cells were discovered as a CD4 T cell subset derived from the thymus and with primary function to reduce conventional T cell activity. However, it became clear that along with Foxp3, Treg cells have the ability to express transcription factors deterministic in T-helper (Th) responses and identity, including Tbet, GATA3 and RORγt^33,34^ (Fig. 1). Gene expression and epigenetic profiling of Treg cells indicate that subsets are closer to undefined Foxp3+ Tregs than they are to their respective Th lineages^35^. Although there is still more to be uncovered regarding the features attributed to Treg subsets, distinct Treg subsets may be able to better orchestrate matching types of immune responses aligned with cell types, such as Th subsets, that express corresponding transcription factor profiles.

Type 1 Tregs express, amongst others, the Th1 cell-associated transcription factor Tbet and have on their surface the chemokine receptor CXCR3^36^. CXCR3 enables strong tropism to tissues in which type 1 responses are ongoing, resulting in the expression of the CXCR3 ligands CXCL9, 10 and 11. Due to their specific recruitment, type 1 Treg cells are thought to be involved in the control of type 1 infections^37^. In their absence, type 1 inflammatory reactions can be enhanced, potentially resulting in tissue damage^38,39^ (Fig. 1).

Type 2 Tregs, characterised by the expression of Th2-associated transcription factors such as GATA3^40^, are enriched in tissues, especially epithelial sites and play a role during inflammation. However, their role has not been closely associated with a type 2 effector response such as allergy or helminth infection, but rather as maintainers of the general Treg population and Foxp3 expression, in order to keep tissue homeostasis at check during infection^41^.

Treg cells express the alarmin IL-33 receptor, ST2^42^. Its expression, especially observed during tissue damage, suggests a role in tissue repair and homeostasis. However, this role seems more complex with ST2 expression not exclusively present on type 2 Treg cells, but found co-expressed with CXCR3 and also CCR6^43,44^ (Fig. 1). This is in line with reports of Gata3, IRF4 and ST2 being required for general Treg cell function and tissue homeostasis^41,45,46^. For example, type 2 Treg cells have been shown to be necessary for the convalescence phase after antibody-mediated renal injury caused by the autoimmune disease crescenting glomerulonephritis. GATA3-expressing Tregs make up over 50% of the Treg cells present, their depletion resulting in increased disease severity. Co-transfer of conventional T cells, including Tregs, into T cell-deficient mice reduced disease severity, while co-transfer with GATA3-deficient Tregs did not suppress disease activity, indicating that type 2-associated gene programme in Treg cells is capable of supressing disease pathology^47^.

Type 3 Tregs are characterised by the expression of RORγt and are particularly present in the colonic lamina propria, where also Th17 cells are enriched^48^. They help control local inflammatory processes against intestinal microbes^49–51^. Type 3 Tregs are important to constrain colonic inflammation, which has an impact on type 3 immunity such as Th17 cells (Fig. 1), their absence resulting in increased IL-17 but also IFNγ production^50^. Induction of colitis results in aggravated disease in the absence of type 3 Treg cells. The response of Treg cells may be microbiota dependent. Although no differences in type 2 cytokines were observed^50^, the absence of type 3 Treg cells can result in the increase of the type 2 anti-helminth response thereby exacerbating type 2 immunity^49^. Type 3 Tregs in the colon are part of a heterogeneous population Treg cell population, with RORγt-expressing Treg cells possibly derived from peripherally generated T cells, indicated by the absence of the transcription factor Helios, under influence of the microbiota. A second population consists mainly of T2 Treg cells, likely thymic derived and expressing Helios, Gata3 and ST2, less sensitive to intestinal microbiota. High Helios levels indicate the capacity to express the alarmin IL-33 and Amphiregulin (Areg), characteristics for a role in tissue-repair^42^, suggesting that a balance between type 3 and 2 Treg cells may be required in tissue repair and homeostasis.

### Treg cell location and migration

Removing type 2 or 3 Treg cells indicates that a strictly linear function of Treg subsets in cross regulating corresponding Th subsets and type of immune responses may be too simplistic. This is further highlighted by the different Treg cell populations found in organs that take on characteristics of the distinct tissues they home to. Treg cells have also been divided in naive and antigen experienced or memory cells in mice and humans based on surface markers and functionality, similar to conventional T cells^52,53^. It has been proposed that mainly antigen experienced Treg cells, sharing markers with effector memory T cells, migrate to inflammatory sites and are found in tissues with tissue specific attributes, with Treg cells showing a gradient in gene expression from a naïve to memory effector status^54,55^.

Muscle harbours a substantial proportion of Treg cells with type 2 characteristics, expressing the IL-33 receptor ST2 and Areg, contributing to tissue homeostasis and regeneration after injury. Absence of ST2 results in reduced Treg cell population size after injury with less muscle regeneration^56^. Resident Treg cells in visceral adipose tissue (VAT) express the peroxisome proliferator-activated receptor (PPAR)γ involved in regulating metabolic genes^43^. These Treg cells represent a high proportion of the total CD4 T cells in young adult mice. Compared to 5–10% representation in lymphoid organs, in VAT Treg cells can reach 40–80% of all CD4 T cells present^57,58^. Several observations point to a preponderant role of VAT Tregs in maintaining VAT metabolic homeostasis^43,59^. Constitutively ablating Treg cells leads to VAT tissue inflammation and disruption of normal metabolic homeostasis, measured by insulin resistance and glucose tolerance tests. Conversely, inducing Treg cell expansion ameliorates the VAT metabolic state, as observed by an increased insulin sensitivity, and consequently decreased fasting blood glucose concentration^43,60,61^. Additionally, genetic excision of PPARγ with help of a Foxp3-Cre PPARγ^fl/fl^ mouse model increases VAT inflammation and deteriorates metabolic health, an effect that can be rescued by treatment with PPARγ agonists^62^.

In addition to a more singular polarised immune response, a coordinated type 1 and type 3 Treg cell response is important for a balanced immune response, such as shown for *Salmonella tiphymurium*. The immune response against this intracellular pathogen comprehends an early Th17 cell response followed by the onset of a potent Th1 cell response, responsible for bacterial clearance. A sequential response from these two immune response types is achieved by the presence and prevalence of different subsets of Treg cells at the site of infection. The more prevalent early type 3 immune response leads to a growing number of type 3 Treg cells being recruited, subsequently enabling a Th1 cell response to clear the bacteria. Depleting Treg cells at different times post-infection, revealed that early depletion of Treg cells prevents a Th17 cell response, while depleting Treg cells at day 6 to 7 post-infection dampens the onset of the critical clearing Th1 cell response, highlighting the preponderance of Treg cells in the balance and success of an immune response^63^. Considering the anti-inflammatory and tissue-homeostatic effect reported for Treg cells at steady state, these are additional beneficial effects beyond active inflammatory responses. Specific Treg cell subsets may be recruited upon acute infection depending on the type of inflammation, but their response is versatile, and their role may shift over time.

### The road to Trm cells

**Fig. 2:**
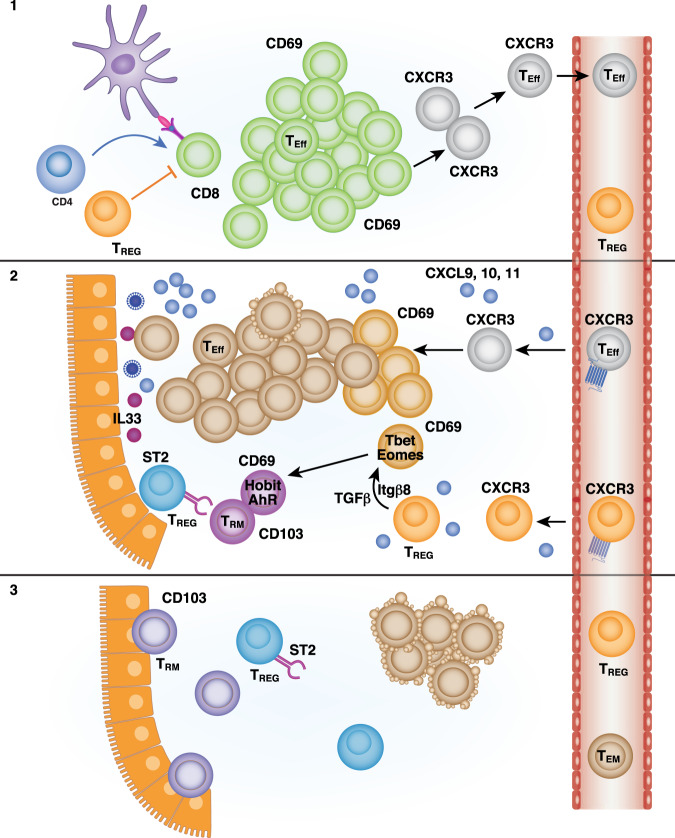
Development of Trm cells and interplay with Treg cells. CD8 T cells are activated in secondary lymphoid organs (SLO) where naïve T cells find their cognate antigen delivered by antigen-presenting cells together with cytokines that determine differentiation (panel 1). Activated CD8 T cells (green) are controlled by CD4 T cells (blue) and Treg cells (orange), and undergo clonal expansion, upregulate CD69 and shed CD62L, eventually leaving the SLO as effector T cells after downregulating CD69. Ongoing inflammation will recruit circulating effector T cells, such as via a CXCL10 gradient attracting type 1 effector CD8 T cells (grey) (panel 2). These cells reencounter antigen and re-express CD69. Many expand and differentiate into fully differentiated effector T cells (colour changing indicating increased terminal differentiation). During the early phase, some effector T cells (expressing Tbet and Eomes) will be in close contact with recruited type 1 Treg cells, also expressing CXCR3. The TGFβ expressed by these cells and its activation via Itgβ8 transforms early effector CD8 T cells into Trm cells (purple) via downregulation of Tbet and Eomes and the expression of Hobit, AhR and CD103. After clearance of the pathogen present most effector T cells undergo apoptosis, with few remaining as circulating Tem or Trm that remain at the original site of infection (panel 3). Treg cells may also remain in the tissues, with a substantial type 2 phenotype with expression of ST2, playing a role in tissue repair and homeostasis.

Once activated in the secondary lymphoid organs, CD8 T cells downregulate CD69 and shed CD62L to join the circulation (Fig. 2). They migrate to the tissues in response to cytokine and chemokine gradients generated by the inflammation response. Effector T cells can be divided in two subsets according to their expression of the IL-7 receptor alpha chain (CD127) and the killer cell lectin-like receptor G1 (KLRG1), a co-inhibitory receptor predominantly expressed on differentiated effector T cells. Short-lived effector cells (SLECs) downregulate CD127 and express high levels of KLRG1, while memory precursor effector cells (MPECs) show the opposite pattern of expression. Trm cells are thought to emerge during the early stages of the immune response in the peripheral lymphoid organs, derived from a cell population in the early stages after T cell activation. While expression of CD127 by Trm cell precursors appears to be generally accepted, the significance of KLRG1 expression is still under discussion. KLRG1 is used as a marker to identify terminally differentiated cells with reduced potential to generate memory cells^64^. Initial studies using adoptive transfers of KLRG1^−^ and KLRG1^+^ effector CD8 T cells concluded only KLRG1^−^ cells were able to generate Trm cells^15^. However, a subsequent study employing a KLRG1-lineage reporting system has challenged this conclusion with the KLRG1^+^ progeny found in memory populations^65^. The lineage-reporting system allows for the identification of a Trm cell-generating population of cells, which initially expresses KLRG1, but loses the expression early post-infection (ex-KLRG1^+^). This provides a possible explanation for the apparent discrepancy, as the initial studies were based on acute KLRG1 protein expression, which would have included ex-KLRG1^+^ cells.

T cell migration can also be modulated by non-chemokines. Trm cells characteristically express inhibitory receptors, such as cytotoxic T-lymphocyte-associated protein (CTLA)-4, lymphocyte-activation gene 3 and programmed cell death protein-1, which have important roles in maintaining T cell tolerance in the tissues in the absence of infection^66,67^. The co-receptor CTLA-4, is particularly intriguing due to its association with the Trm cell phenotype. One of the more promising avenues in cancer therapy consists in the blockade of these immune checkpoints using neutralising antibodies^68^, which allows T cells to fight tumour cells more effectively. CTLA-4 is immediately upregulated upon T cell activation with expression peaking 48–72 h later and waning after that^69^. It acts by outcompeting another T cell co-receptor, CD28, in binding their common ligands CD80 and CD86 on the surface of antigen-presenting cells (APC). CD28 interaction with these ligands provides a second signal for T cell activation and blocking the second signal results in a higher activation threshold for T cells. The reverse-stop signal model proposed in 2008 puts forward that CTLA-4, by limiting T cell – APC interaction, reduces T cell dwell time, modulating TCR signalling intensity and simultaneously inducing cell polarisation and motility^70^. Taking into account the kinetics of CTLA-4 expression as well as the restricted timing for T cell migration into the tissues^71^, it is tempting to speculate on a possible connection between the two events. It is plausible that CTLA-4 expression serves the dual purpose of selecting higher affinity T cells and increasing migration capacity, placing the most effective T cells in the tissues.

### Shaping the Trm cell response

To access the tissues, T cells must arrest on the endothelial surface and cross the endothelial wall. This involves the engagement of molecules expressed on both T cells and endothelial cells, such as LFA-1 which binds to ICAM-1 on endothelial cells, the integrin α4β1, which binds to VCAM-1, and α4β7, which mediates T cell binding to the mucosal adhesion molecule-1 (MAdCAM-1) in the gut. Finally, there are also indications that CTLA-4 might be involved in transendothelial migration, facilitating access to the tissues by adhering to CD86 expressed on endothelial cells^72^.

Access to the inflamed tissue is a requirement for Trm cell development. There will be tissue-specific factors involved such as CCR9 and CCR8 for the intestine and skin respectively, but also in part the type 1-immunity associated chemokine receptor CXCR3 during type 1 infections^15^ (Fig. 2). Once inside the tissue, effector T cells re-express CD69, preventing egress, which can be followed by CD103. Both molecules, but especially CD69, play an important role in ensuring Trm cell persistence in the tissues^15^. TGFβ is essential for the upregulation of CD103 in Trm cells and many cell types such as immune cells, epithelial cells and cancer cells secrete it. It not only induces CD103 expression, but it is also involved in the downregulation of two transcription factors key in the Trm maturation process – Tbet and Eomes. While Eomes expression is completely abrogated in mature Trm cells, residual levels of Tbet are maintained in order to ensure the expression of the IL-15 receptor, signalling through which is important for Trm cell survival^20^.

However, TGFβ is produced as an inactive precursor requiring activation before the signalling cascade can be initiated. This constraint allows for a tight regulation of TGFβ availability in the local microenvironment. TGFβ-activating pathways are not yet completely understood, but several players have been identified, including αv-integrins. The αvβ8 integrin, expressed on epithelial and Treg cells is able to activate TGFβ and plays an important role in Trm cell development^9,73–75^ (Fig. 2). Although the role of Treg cells in modulating the T cell response towards the end of an immune reaction is well known, Treg cells are present in inflammatory sites from early on after the initial recruitment of T cells^76^. Treg cells, like effector T cells, are recruited in response to tissue damage and stress signals, and are involved in tissue repair^77^. As previously discussed, all Treg cells may have the capacity to contribute to tissue repair, with a more type 2 immunity phenotype.

### Trm cell metabolism

Upon TCR ligation, naive CD8^+^ T cells employ a metabolic programme via transcriptional and translational means that enable rapid cell division and differentiation into effector T cells. Furthermore, effector T cells require sufficient energy to migrate through the body in search of their antigen. After receiving required activation signals, kinases interact to form a complex, also intersecting with and altering metabolic pathways^78^. The presence of pyruvate dehydrogenase in the mitochondrial membrane is reduced, thereby increasing the use of pyruvate, obtained via glycolysis, for cytosolic oxidation to lactate and limiting access to pyruvate for mitochondrial oxidative phosphorylation (OXPHOS). Glycolysis is gradually increased and sustained after co-stimulation signalling^79^. There are several aspects that are not well understood since the net effect is reduced generation of ATP while the uptake of glucose is initially not increased^80^.

It takes ~24-h for CD8 T cells to start the first cell division, which then initiates the transcriptional programmes to facilitate nutrient uptake, primarily sugars, amino acids and fatty acids^78,81,82^. Subsequently, uptake of metabolites is increased, eventually resulting in cytotoxic function and cytokine production^83–85^. Metabolite availability and the T cell demand are strictly regulated by metabolic checkpoints, regulating proliferation, function and survival^86^, using both glycolysis and OXPHOS^87,88^. It has been reported that prior to the first cell division, the fate of the daughter cells is determined by asymmetric distribution of MYC and mTORC1^89,90^. Daughter cells proximal to the TCR receive more MYC and mTORC1 content that results in higher glycolytic activity and short-lived effector cells. In contrast, lower levels of MYC and mTORC1 reduces glycolytic activity, resulting in long-term cell survival.

In vivo, CD8 memory T cells show reduced lipid uptake compared with effector T cells^91^. Although in vitro experiments indicate a potential role for lipids, genetic deletion of the enzyme that transports enzymes into the mitochondria, carnitine palmitoyltransferase (CPT)1α or OXPHOS function does not prevent the generation of memory T cells^92,93^. This may indicate T cells can adapt under different circumstances and have metabolic flexibility. Of additional interest is the use by memory CD8 T cells of gluconeogenesis and storage of energy in the form of glycogen^94^, presumably to ensure rapid proliferative capacity upon reactivation.

Trm cells, especially those at epithelial barriers, the intestine and skin, contain many effector molecules and are poised for activation^26,29,31,95^. Intriguingly, these cells do not show the increased OXPHOS capacity reported for circulating memory T cells, but appear to be arrested in their semi-activated state^31,96,97^. The cells are phenotypically similar to effector cells, such as expression of early activation markers, notably CD69. They express high levels of cytotoxic molecules such as granzymes, while they do not show evidence of cell proliferation or cytokine secretion, but this can be rapidly induced^6^. Metabolic assays indicate that Trm cells do not increase their metabolic function, OXPHOS as well as glycolysis, when assessed after TCR triggered activation. This can in part be explained by the altered make-up of the mitochondrial lipids, the cardiolipins, responsible for anchoring and assembly of the electron transport chain complexes and shaping the membrane^31^. Cardiolipins are important in mitochondrial function and in T cell activation^98^, but their composition of long or shorter chains and level of saturation is directly linked with Trm cell activation^31^. Although transcriptional activity of enzymes in lipid metabolism are increased in Trm cells and the storage of lipids in droplets is apparent^31,96^, how much of this fuels cell metabolism or is used for effector functions, such as eicosanoids, remains unknown. Of interest is the differential expression of fatty acid-binding proteins (FABP), which are expressed in a tissue-specific manner on immune cells, including Trm cells, and seem imposed by the host organ^99^. Skin Trm cells, which similarly contain lipid droplets and express FABP4 and 5, have been reported to take up lipids in vitro, but the effect on OXPHOS was limited^96^. Genetic deletion of FABPs, 4 and 5 for the skin or 1 for the liver, reduced the number of Trm cells present^96,99^. However, lipids or altered cardiolipins are only part of the altered metabolism in Trm cells, since glycolysis levels at the times tested after activation also remain low compared to circulating memory CD8 T cells.

### Interplay of Trm and Treg cells

Trm cells, at least in the barrier tissues, show a partial activation state with characteristics in surface markers, effector molecules and metabolic capacity that are distinct from naïve, effector and other memory T cell populations^31,95,100^. The arrest in this semi-activation state may partially be explained by an altered metabolism. The biological reason is not yet clear, but may relate with the long-term survival and functional requirements of Trm cells. An important outstanding question is how Trm cells develop. There are indications that the tissue migratory capacity of effector cells is time-limited, with virus specific CD8 T cells being able to migrate to the intestinal IEL compartment at 4.5 days after infection, but losing this capacity by day 7^71^. This allows for a limited window of time for Trm cell generation, pointing to a biological significance in restricting generation of Trm cell precursors to the initial phase of the immune response. This may link with the initial cell divisions taking place after release from the SLOs^89,90^, and with the metabolic status of the early effector cells as well as the requirements for maintaining Trm cells in a semi-activated state^31^. Furthermore, Trm cells, at barrier sites based on their expression of CD69 and CD103, as well as the absence of KLRG-1 and Eomes, are found early during infection at the site of inflammation during an ongoing inflammatory response^9,15^. This suggests that Trm cells develop very early during the initial infection stage and are derived from not fully differentiated effector T cells.

An infection and rapid activation of innate pathogen recognition receptors results not only in identification of the invader and early immune response to contain it, but also in the production of chemokines in accordance with the identity of the pathogen. Accordingly, intracellular pathogens trigger a type 1 response, involving the secretion of the chemokine CXCL-10, attracting CXCR3-expressing cells (Fig. 2). Type 2 responses against helminths and type 3 responses against extracellular pathogens would follow a similar pattern with secretion of respective chemokines. Inflammation results in a rapid influx of effector cells, accompanied by Treg cells which remain at a remarkably stable ratio with CD4 effector T cells^9,63^. This is not surprising since the role of Treg cells is to maintain a balanced response, to reduce the threat of the invading pathogen while preserving the integrity of the tissues and the optimal functioning of the host. Furthermore, Treg cells play a role in immune resolution and tissue repair^101^. Uncontrolled inflammation can result in tissue damage, impaired healing, with potential long-term tissue remodelling and reduced function. Treg cells are known to affect T cells, particularly CD8 T cells during the effector stage, reducing their functional prowess^102,103^.

We have recently shown that Treg cells also play an important role in the generation of Trm cells through their ability in making TGFβ bio-available. Treg cells are recruited together with their CD4 effector T cell counter-parts. Both express CXCR3 and migrate to sites of type 1 infection where CXCR3 ligands are secreted early during inflammation^9^. This is particularly interesting, as Treg cells are mostly known for their suppressive function, controlling other cell populations and preventing excessive inflammation. The response to an intracellular pathogen results in the recruitment of primarily type 1 Treg cells, expressing Tbet and the chemokine receptor CXCR3. These Treg cells are located in the vicinity of effector CD8 T cells^9,39^ (Fig. 2). This provides opportunity to convert pre-TGFβ into bioactive TGFβ with the help of Integrinβ8, resulting in upregulation of CD103 and Trm cell development^9,73^. These data suggest that Trm cell differentiation occurs early during infection and at the site of inflammation.

Recruiting Treg cells to the right place at the right time seems important^104^, in absence of CXCR3-expressing Treg cells, Trm cell development is much reduced^9^. Although TGFβ is important, its timed delivery may be equally so. The number of cell divisions of effector T cells is deterministic of their function^105^. The proliferative strength is determined by TCR and co-receptor stimuli as well as cytokines. Treg cells can oppose this by reducing co-stimulatory signals via CTLA-4 expression and the anti-proliferative action of TGFβ, thereby limiting cell division and differentiation^106,107^. In addition, Treg cells can inhibit the CD8 effector response via metabolic inhibition. The expression of the high affinity IL-2 receptor will reduce T cell growth^108^. Treg have high concentrations of cAMP, which by cellular transfer can inhibit the activity of effector T cells and antigen-presenting cells^109^. In addition, Treg cells express CD39 and CD73, which reduce stimulation and alter T cell metabolism. CD39 converts ATP and ADP into AMP, with CD73 converting AMP to adenosine^110^. Adenosine binding to the A2 adenosine receptor results in metabolic inhibition of T and dendritic cells. Furthermore, expression of PD-1 and CTLA-4 alter the immune response, while CD36 enables VAT Tregs to scavenge fatty acids from the environment. This provides Treg cells with tools to dampen the effector CD8 T cell response and enhance Trm cell development^111^, potentially required to initiate their arrest in a poised but semi state of activation.

The contribution of Treg cells to the generation of T cell memory thus adds another layer to their functionality, one that ultimately results in a swift and more efficient immune response upon reinfection, thereby reducing the risk of immunopathology. Initial subversion of early effector cells entering inflamed tissues may dampen the anti-microbe response, but it may thereby prevent immunopathology and strengthen future protection against invasion.

### The benefits and dangers of Trm cells

Although Trm cells have a beneficial role during microbial challenge (reviewed here^112^), the generation of memory T cells, especially at barrier sites occupied by a beneficial microbiota may have negative consequences. Aberrant generation of Trm cells may contribute to chronic inflammation, the development of autoimmune and allergic conditions. Although reports are still rare, Hondowicz et al. identified a lung resident T cell population which induced asthma in mice after house dust mite antigen exposure^113^. Furthermore, the use of checkpoint inhibitor therapy in melanoma patients has been linked to increased development of vitiligo and Trm cells are enriched in skin lesions of vitiligo patients^114,115^. Their cytotoxic potential and persistence at the site of lesions suggest a role as active players in disease. Similarly, Trm cells maintained in the small intestine contribute to coeliac disease, triggered upon re-encountering gluten^116^. This reactivation of Trm cells is likely to have broader implications when inappropriate responses against luminal antigens are launched and Trm cells generated, contributing to chronic inflammatory diseases^117^.

In the last few years, Trm cells have also emerged as influential players in the field of tumour immunology. Experiments using animal models^118–120^ as well as observational studies with human patients have revealed that these cells enhance anti-tumour immunity and are associated with better clinical outcomes. Accumulation of CD8^+^ CD103^+^ Trm cells in tumour tissue is strongly correlated with improved patient survival in several types of cancer, such as breast^121^, lung^122^, ovarian^123^ and melanoma^124^. Trm cells are characterised by the expression of several molecules that play a role in their function and thus offer potential, through their manipulation, to enhance immune therapy.

Expression of CD103 by CD8^+^ Trm cells in tumours has been specifically linked to favourable disease progression. Indeed, in a lung tumour model it was shown to facilitate antigen recognition and to induce T cell effector function in synergy with TCR engagement^125^. Trm cells also confer increased protection against infection. For example, skin Trm cells protect against reinfection with herpes simplex virus (HSV) after acute infection and Trm cells in the nasal tissue block the transmission of influenza virus from the upper respiratory track to the lungs^126,127^. In the liver, Trm cells were shown to protect against liver-stage malaria in a murine model and to be associated with disease control in humans infected with hepatitis B virus^30,128^.

This creates the intriguing question of whether Treg cells, generally regarded as enhancing tumour progression and inhibiting anti-tumour responses, can play a beneficial role. Currently there is no data. However, an anti-tumour response relies on a cytotoxic type 1 response. Detailed investigation of breast Treg populations found CCR8-expressing Treg cells, a chemokine receptor associated with type 2 immune responses, to be strongly associated with poor tumour prognosis^129,130^. This suggests that the type of Treg subset recruited or present in a tissue may impact the generation of Trm cells and the reactivity of CD8 T cells, but may particularly highlight that the Treg cell repair response is anti-tumour inhibitory^131,132^.

### Concluding remarks and remaining questions

The role of Treg cells, recruited during the early stage of inflammation is within their well-established role of safeguarding immune responses from immunopathology. Initial influx of effector CD8 T cells will be blunted, preventing tissue damage and aberrant responses^38^. Importantly, their role in the seeding of Trm cells is important in subsequent encounter with pathogens, strengthening the first line of defence, enabling swift pathogen clearance or at least a reduction in pathogen load, thereby preventing reinfection, resolving infection locally and reducing the amplitude of subsequent immune responses required^76^. We hypothesise the different subsets of Treg cells are recruited to the site of infection, depending on the type of infection ongoing, matching chemokine ligands and receptors but that these subsets do not necessarily have specific functions. The type of immune response is not fixed in time and may swiftly convert in the initial stages of infection, such as the switch from IFNγ to IL-4 production during helminth infection and IL-17 to IFNγ production during bacterial and fungal infections^48,133^. The recruitment of specific subsets and if specific functions are associated with these or with functionality changes over time remain to be determined.

Treg cell-facilitated and TGFβ-mediated expression of CD103 at epithelial sites^9,73,134^, is one event important to establish a Trm cell population. Re-expression of CD69 is most likely due to reencounter with cognate antigen, responsible for the establishment and retention of a Trm cell population at the specific site of infection^7,12,135^. We hypothesise that the establishment of Trm cells from early effector cells has functional consequences. The number of times cells divide has been shown as a critical component of T cell responses^136^, with a strong correlation of magnitude of cytotoxic response and Trm cell presence^122^. This is in line with the clinical outcome in tumours, infection challenges and with the functional characteristics of Trm cells that appear in a semi-activation state as if arrested effector T cells^31^. How this state is induced remains an open question. However, this poised state suggests rapid effector potential, important in the first line of defence against invading microorganisms. Hence, the control of this altered activation state, at least in part determined by metabolic pathways, and the stimuli required resulting in full effector potential remain important areas of study. This is especially relevant in the design and use of vaccines, prevention of unintentional Trm cell activity, and could be directly applicable to immunotherapies against solid tumours.
